# Isolation, identification and complete genome sequence analysis of a strain of foot-and-mouth disease virus serotype Asia1 from pigs in southwest of China

**DOI:** 10.1186/1743-422X-8-175

**Published:** 2011-04-16

**Authors:** Xin Yang, Ying-shun Zhou, Hong-ning Wang, Yi Zhang, Kun Wei, Ting Wang

**Affiliations:** 1 School of Life science, Sichuan University, Animal Disease Prevention and Food Safety Key Laboratory of Sichuan Province," 985 Project" Science Innovative Platform for Resource and environment Protection of Southwestern, Key Laboratory of Bio-resources and Eco-environment, Ministry of Education, Chengdu, Sichuan, 610064, China

## Abstract

**Backgroud:**

Foot-and-mouth disease virus (FMDV) serotype Asia1 generally infects cattle and sheep, while its infection of pigs is rarely reported. In 2005-2007, FMD outbreaks caused by Asia1 type occurred in many regions of China, as well as some parts of East Asia countries. During the outbreaks, there was not any report that pigs were found to be clinically infected.

**Results:**

In this study, a strain of FMDV that isolated from pigs was identified as serotype Asia1, and designated as "Asia1/WHN/CHA/06". To investigate the genomic feature of the strain, complete genome of Asia1/WHN/CHA/06 was sequenced and compared with sequences of other FMDVs by phylogenetic and recombination analysis. The complete genome of Asia1/WHN/CHA/06 was 8161 nucleotides (nt) in length, and was closer to JS/CHA/05 than to all other strains. Potential recombination events associated with Asia1/WHN/CHA/06 were found between JS/CHA/05 and HNK/CHA/05 strains with partial 3B and 3C fragments.

**Conclusion:**

This is the first report of the isolation and identification of a strain of FMDV type Asia1 from naturally infected pigs. The Asia1/WHN/CHA/06 strain may evolve from the recombination of JS/CHA/05 and HNK/CHA/05 strains.

## Background

Foot-and-mouth Disease is a highly contagious and economically important disease of cloven-hoofed animals, predominantly for cattle, sheep, and pigs. The aetiological agent, foot-and-mouth disease virus, is classified as small icosahedral virus of the *Aphthovirus *group within the Picornaviridae family. There are seven immunologically distinct serotypes of the virus, namely types O, A, C, SAT1, SAT2, SAT3 and Asia1, and subtypes have also been found within some serotypes [1].

FMD serotype Asia1 has been epidemic in China for more than 50 years. This serotype usually infects cattle and sheep, and its infection of pigs is seldom reported [2]. In 2005-2007, FMD outbreaks caused by Asia1 type occurred in many regions of China, as well as some parts of East Asia countries. During the outbreaks, there was not any report that pigs were found to be clinically infected [2].

FMDV has a single-stranded positive sense RNA genome of approximately 8.5 kb in length, including the 5' untranslated region (5'UTR), a large singe open reading frame (ORF), and the 3' untranslated region (3'UTR) [3,4]. The 5' UTR consists of a short (S) fragment, a poly (C) tract, and a long fragment (5'LF-UTR), which contains three or four tandemly repeated pseudoknots (PKs) and an internal ribosome entry site (IRES) [5]. The ORF encodes a polyprotein that can be cleaved to form four structural proteins (VP4, VP2, VP3 and VP1) and 8 non-structural proteins (L, 2A, 2B, 2C, 3A, 3B, 3C and 3D) [5]. The VP1 protein plays an important role in virus attachment, protective immunity and serotype specificity, and nucleotide sequencing of this region has been extensively used for molecular epidemiology studies on FMD [6,7]. The G-H loop of the VP1 protein of FMDV spanning residues 134-158 contains conserved Arg-Gly-Asp (RGD) tripeptide, which is considered to be a ligand for cell-surface attachment [8]. In addition, FMDV 3A region has been implicated in virus virulence and host range, similar to the 3A proteins of other picornaviruses [9]. The 3'UTR of about 90 residues with a poly (A) tail (35-100nt) at 3'-end is likely to be a site of interaction with viral and host proteins for RNA replication [10,11].

This study, for the first time to our knowledge, described the isolation and identification of a strain of FMDV type Asia1 from pigs in China. To investigate the genomic feature of the strain and further understand its role in epidemiology of FMDV, the complete genome of the strain was sequenced and compared with sequences of other strains of FMDV Asia1 by phylogenetic and recombination analysis.

## Materials and methods

### Sample collection and clinical samples treatment

Three samples of ruptured vesicular fluids were collected from FMD-suspected pigs in a pig farm in southwest of China in 2006. The samples were transported from the collection site to diagnostic laboratory in 0.04 M phosphate buffer (pH 7.2) with 50% glycerol at 4°C and stored at -20°C until tested.

### Virus isolation and identification

Established cell layer of BHK-21 cells were inoculated with 0.2 ml the three samples of vesicular fluids, respectively. The cell cultures would be examined for Cytopathic effects (CPE) for 48 hours. If CPE was formed, the cells would be collected for subsequent experiments. If no CPE was detected, the cells would be frozen and thawed, and used to inoculate fresh cultures of 0.2 ml and examined for CPE for another 48 hours. The infected BHK-21 monolayer cells were subjected to three freeze-thaw cycles to release the viral particles. The viral suspension was clarified from the cell debris by centrifugation at 800 × g for 10 min and stored at -70°C for the following experiments.

The FMDV O, Asia1 and A type positive serums were chosen as antiserum in complement fixation test (CFT). The CFT was performed by adding 0.2 ml virus sample, each of the 3 type of antiserum and complement in tubes, respectively. After incubating the mixtures at 37°C for 1.5 h, sensitized sheep erythrocytes was added to each tube, and the mixtures were incubated for 1 h at 37°C. Degrees of hemolysis were determined by visual reading. The tests were set up in triplicate. Also, virus sample, antisera, complement, and erythrocytes controls were set up with each test.

### RNA extraction and RT-PCR

The RNA was extracted from the infected cell culture supernatant by using RNeasy kit (Qiagen). The cDNA synthesis was carried out using Random primer_9 _and Oligo (dT)_18_, and Superscript П reverse transcriptase (Invitrogen). The cDNA fragments were amplified by PCR (Biorad) using LA Taq DNA polymerase (TaKaRa, Japan a) and seven pairs of primers (Table 1). The 5'RACE was performed using 5'-full RACE kit (TaKaRa, Japan) following the manufacturer's instructions. The cDNA fragment at the 3' UTR was amplified by the 3'rapid amplification of cDNA ends (3'-RACE) method. The PCR parameters included an initial denaturation at 94°C for 2 min, followed by 30 cycles of denaturation at 94°C for 50 s, annealing 58°C for 50 s, and extension at 72°C for 1-3 min depending on the sizes of the products and a final extension at 72°C for 10 min. The 5 μl PCR products were electrophoresed in a 0.8% agarose gel containing ethidium bromide, and visualized under UV light.

**Table 1:** Synthetic oligonucleotides used for amplification of the FMDV genome^a^

Primers	Position	Sequence(5'-3')	Fragment
S1 ^b^	Used for 5'-RACE	CATGGCTACATGCTGACAGCCTA	5'RACE
S2	344-367	TGAAAGGCGGGCGCTTGGTGACA	5'RACE
U1	379-399	TAAgTTTTACCGTCGTTCCC	UFR
U2	718-736	ACCGAGCGTGGAGTCAAT	UFR
A1	524-541	CGGAAGTAAAACGGCACA	A
A2	2276-2293	GATTTCCAACAGCGGTCA	A
B1	2217-2233	CGTGTTTGGCAGCCTCAT	B
B2	4700-4717	CCACGGGTTCAGGTCTCG	B
C1	4576-4593	AACGGCCCAAGCAAGTAT	C
C2	6599-6616	TGGAAACGCACGAGCAGT	C
D1	6205-6222	GGCAGAGCCATGACAGAC	D
D2	7458-7475	GGGTGGAAGCCAAACTCT	D
GSP1	7446-7463	GGTGTTTCGCACAGAGTT	3'RACE
R1	3'end	TAAgCAAgCATgCCATATgTT	3'RACE
3' RACERT^c^	Used for 3'-RACE	TAAgCAAgCATgCCATATg(T)_15_	

^a ^The primers used in this study were designed based on JS/CHA/05 (Accession NO. EF149009)

^b ^S1 was used in 5'-RACE for amplification of the 5' end of RNA.

^c ^3'RACERT was used in 3'-RACE for amplification of the 3' end of RNA.

### Genome sequencing

The target fragment of PCR products were purified using Gel Extraction Kit (OMEGA bio-tek). The purified products were ligated to pMD18-T vector (TaKaRa, Japan) and transfected into *E. coli *JM109. The positive clones were selected to provide for sequencing by Shanghai Sangon Biological Engineering Technology and Services Co., Ltd. Each nucleotide was determined from three identical results. Sequences were assembled into complete genome sequence using SeqMan || program of DNAstar software package (DNAstar Inc., USA)

### Sequence comparison and phylogenetic analysis

Ten complete genome and thirty-three VP1 gene sequences of FMDV Asia1 type isolates of bovine origin and three FMDV O type (as out group) from GenBank were involved in the analysis (Table 2). Multiple sequence comparisons at the nucleotide and the amino acid level were performed by MegAlign program of DNASTAR package. The phylogenetic tree based on VP1 sequences was constructed by using the program MEGA 4.0 [12] with Kimura 2-parameter nucleotide substitution model. The bootstrap values were determined from 1000 replicates of the original data.

**Table 2:** Reference FMDV strains selected from GenBank

**Genbank Accession no**.	Strain	Species of origin	Country and year of isolation	**Genbank Accession no**.	Strain	Species of origin	Country and year of isolation
FJ906802	**WHN/CHA/06**	**porcine**	**China, 2006**	EF187273	HB/CHA/05	bovine	China, 2005
EF149009	**JS/CHA/05**	**bovine**	**China, 2005**	EF187272	QH/CHA/05	bovine	China, 2005
AY593797	**3kimron/61**	**bovine**	**Israel, 1963**	EF185303	BJ/CHA/05	bovine	China, 2005
EF457988	**AFG/03**	**bovine**	**Afghanistan, 2003**	EU091347	MYA/06	bovine	China, 2006
DQ533483	**ZB/CHA/58**	**bovine**	**China, 1958**	EU667461	LAO/3/98	bovine	Laos, 1998
EF149010	**HNK/CHA/05**	**bovine**	**Hong Kong, 2005**	FJ785294	IND/147/04	bovine	India, 2004
NC004915	**India/72**	**bovine**	**India, 1972**	FJ785235	HKN/3/05	bovine	Hong Kong, 2005
AY687333	**IND/321/01**	**bovine**	**India, 2001**	FJ785259	NKR/2/07	bovine	South Korea,2007
AY390432	**YNBS/58**	**bovine**	**China, 1958**	FJ785283	VIT/8/06	bovine	Viet Nam, 2005
AY593795	**PAK/1/54**	**bovine**	**Pakistan, 1954**	FJ785284	VIT/1/06	bovine	Viet Nam, 2005
AY593800	**LEB/83**	**?**	**Lebanon, 1983**	DQ101238	India/04	bovine	India,2004
FJ785252	MOG/05	bovine	Mongolia, 2005	EU667460	Laos/96	bovine	Laos, 1996
FJ785240	HKN/8/05	bovine	Hong Kong, 2005	FJ785268	Pry/RUS/05	bovine	Russia,2005
FJ785229	CAM/5/97	bovine	Cambodia, 1997	FJ785267	Kha/RUS/05	bovine	Russia, 2005
FJ785290	VIT/4/06	water buffalo	Viet Nam, 2005	FJ785266	PAK/22/05	bovine	Pakistan, 2005
DQ101242	India/02	bovine	Afghanistan, 2001	FJ785228	CAM/9/80	?	Cambodia, 1980
FJ785227	AFG/3//01	bovine	Afghanistan, 2001	FJ785249	KRG/2/04	?	Kyrgyzstan, 2004
FJ785246	IRN/30/04	?	Iran, 2004	DQ121116	IND/80	?	India,1980
FJ785258	MYA/1/05	?	Myanmar,2005	DQ121117	India/81	?	India, 1981
FJ785277	UZB/2003	?	Uzbekistan, 2003	AF308157	O/TW/97	porcine	Taiwan, 2002
DQ121401	Russia/05	?	Russia, 2005	AY317098	O/HKN/02	?	Hong Kong, 2002
EF185304	GS/CHA/05	bovine	China, 2005	AY312589	O/SKR/02	porcine	South Korea,2002

^a ^Eleven completely sequenced FMDV strains are indicated in boldface.

^b ^Question mark (?) indicates that data is inadequate.

^c^All the reference strains, except O/TW/97, O/HKN/02, and O/SKR/02, are FMDV serotype Asia1.

### Putative recombination analysis

The complete genome sequences based on the multiple alignment result were used for recombination analysis with the SimPlot program version 3.5.1[13]. Similarity plot were built using Asia1/WHN/CHA/06 as the query sequence against the complete genomic sequences of 8 strains of serotype Asia1. To further investigate the potential recombination event, the Recombination Detection Program (RDP) [14], GENECONV [15], BOOTSCAN [16], and MaxChi [17] methods were carried to employ Recombination Detection Program 3 (RDP3) [18] software. Statistical significance was set at the P = 0.05 level. Each analysis was conducted twice to ensure repeatability of results.

## Results

### Virus isolation and identification

Virus from one sample was adapted to BHK-21 cells and CPE occurred when inoculated fresh cultures first time after 24 hours, but the other two didn't show any CPE by blind passage 5 times. Therefore, one strain of virus was isolated, and the first passage of the strain in BHK cells was used for following experiments. For complement fixation test, hemolysis occurred in the mixtures containing FMDV O and A type positive serums, whereas the mixtures containing FMDV Asia1 type positive serums didn't show any virtual change. Thus, the isolate was identified as FMDV of serotype Asia1, and was designated as Asia1/WHN/CHA/06.

### RT-PCR and complete genome of Asia1/WHN/CHA/06

In order to determine the full-length genome sequence of Asia1/WHN/CHA/06 strain, the seven RT-PCR cDNA amplicons covering the entire RNA genome were cloned and each nucleotide was determined from three identical results. The genome sequence was submitted to GenBank with the accession number of FJ906802. The complete genome sequence of Asia1/WHN/CHA/06, excluding poly(C) and poly(A) tail, is 8161 nt in length, containing an ORF, a 5' UTR, and a 3' UTR. ORF is 6990 nt (encoding 2329 aa)in length, which consists of L (603 nt), P1 (2199 nt), P2 (1464 nt), and P3 (2724 nt) genes. P1 protein is predicted to be cleaved into four structural proteins, including VP4 (85 aa), VP2 (219 aa), VP3 (218 aa), and VP1 (211 aa). There are three non-structural proteins in P2, including 2A (16 aa), 2B (154 aa), and 2C (318 aa). P3 protein contains 3A (153 aa), 3B (71 aa), 3C (213 aa), and 3D (471 aa) four proteins. The 5' UTR consists of S, FUR, and IRES structures, which is 366, 258, and 454 nt in length, respectively. The 3' UTR of Asia1/WHN/CHA/06 was 93 nt in length, followed by a 66 bp poly (A) tail at least.

### Sequence analysis

Nucleotide and amino acid identity of different regions of Asia1/WHN/CHA/06 genome were compared with other six FMDV Asia1 strains (Table 3). Asia1/WHN/CHA/06 genome exhibited identities ranging from 90.2 to 98.5%, and the highest similarity with JS/CHA/05 with identity rate of 98.5%.

**Table 3:** Similarity comparison of nucleotide and amino acid sequences of Asia1/WHN/CHA/06 to other FMDV Asia1 type isolates

Asia1/WHN/CHA/06	Nucleotide (amino acid) sequence similarity (%) with Asia1/WHN/CHA/06					
	
	JS/CHA/05	ZB/CHA/58	HNK/CHA/05	IND/321/01	YNBS/58	PAK/1/54
**5'UTR**	**96.8**	86.3	84.0	86.7	85.8	84.4
**S**	95.1	83.3	77.9	83.3	83.3	78.7
**5'LF-UTR**	98.3	88.5	87.8	89.0	89.0	88.1
FUR	96.5	87.6	88.4	89.9	88.4	88.8
**IRES**	**99.3**	89.4	89.0	89.0	86.8	88.5
ORF	98.5 (97.7)	89.8 (94.9)	89.7 (95.7)	89.6 (95.5)	89.7 (95.1)	89.6 (96.3)
L	98.3 (98.5)	84.2 (90.0)	87.7 (95.0)	86.2 (92.5)	83.9 (89.6)	86.7 (96.0)
VP4	99.2 (100.0)	89.8 (97.6)	91.8 (100.0)	90.2(100.0)	89.0 (96.5)	92.2 (98.8)
VP2	98.9 (98.6)	87.4 (92.7)	87.4 (95.0)	86.1 (94.5)	87.5 (93.2)	86.5 (95.4)
VP3	99.7 (100.0)	88.8 (95.9)	86.9 (96.8)	84.4 (96.3)	88.5 (95.9)	87.2 (96.8)
**VP1**	98.9 (99.1)	85.5 (93.4)	82,1 (89.6)	81.4 (87.7)	84.2 (91.5)	84.0 (87.7)
2A	100.0(100.0)	83.3 (93.8)	95.8 (100.0)	89.6(100.0)	85.6(100.0)	95.8(100.0)
2B	98.5 (98.7)	93.3 (96.1)	93.3 (96.8)	94.4 (97.4)	94.2 (97.4)	91.3 (98.7)
2C	96.6 (97.2)	93.1 (97.2)	91.7 (95.9)	93.1 (96.9)	92.3 (95.9)	92.6 (97.2)
**3A**	98.7 (98.0)	89.1 (89.5)	89.5 (93.5)	91.3 (94.1)	90.8 (92.8)	88.9 (92.8)
**3B**	**93.4 (95.8)**	92.5 (93.0)	**97.7 (100.0)**	93.4 (97.2)	92.5 (93.0)	93.4 (98.6)
3C	97.5 (99.5)	90.1 (97.2)	92.5 (97.7)	92.5 (97.7)	90.1 (96.7)	90.6 (98.6)
3D	99.1 (99.1)	93.2 (98.1)	93.5 (98.1)	92.6 (97.0)	93.0 (98.1)	93.1 (98.1)
**3'UTR**	87.1	81.7	87.1	74.2	76.3	87.1
**Complete**	**98.5**	90.5	90.3	90.2	90.5	90.3

^a ^The interests of the names of the fragments and data are indicated in boldface.

^b ^Similarity rates of amino acid are shown in brackets.

The ORF of Asia1/WHN/CHA/06 was 89.6 - 98.5% identical with the six reference sequences at the nucleotide level with no deletion or insertion. Compared with 40 sequences of VP1 genes, Asia1/WHN/CHA/06 exhibited identities ranging from 81.0 to 99.2% (data not shown). By comparing the 40 amino acid sequences of G-H loop region in VP1 protein, Asia1/WHN/CHA/06 got highly consistency with the stains isolated after the year of 2004, and no mutation was observed in RGD tripeptide. Interestingly, RGD mutants (RGD→HGD) were examined in Asia1/3kimron/61 and KRG/2/04. For bordering region of RGD of Asia1/WHN/CHA/06, there was no amino acids' mutation at RGD +1 and +4 sides, but a mutation at the RGD +9 (N→D). The 3A gene of Asia1/WHN/CHA/06 strain was 459 nt without insertions and deletions at this region. However, three amino acids' mutations occurred at the residues 86 (D→N), 93 (I→N), and 131 (H→R). Interestingly, Asia1/WHN/CHA/06 displayed the highest nucleotide similarity with JS/CHA/05 at all the regions except 3B gene (93.4%), whereas it showed the highest similarity (97.7%) with HNK/CHA/05 at 3B gene.

The 5' UTR of Asia1/WHN/CHA/06 shared 96.8% identity with the JS/CHA/05 strain, whereas it showed only 84.4-86.7% identity with other 5 reference strains. For the S fragment of 5'UTR, Asia1/WHN/CHA/06 displayed 3 nucleotide deletions located at nt 72, 74, and 184. The 712-nt of Asia1/WHN/CHA/06 of 5' LF-UTRs was observed, whereas within other reference strains, the length of 5' LF-UTRs ranged from 690 nts to 714 nts. Asia1/WHN/CHA/06 strain showed four PKs structure in 5' LF-UTR, which was the same as other reference isolates, and no nucleotide deletion was observed. For the IRES fragment of 5' UTR, Asia1/WHN/CHA/06 was more genetic conservative than that of S fragment, displaying 86.8-99.3% homology to other Asia1 type isolates, and only found to be 2 nucleotide deletions. The sequence comparison of the 3' UTR regions showed that Asia1/WHN/CHA/06 exhibited 74.2-87.1% homology to other six Asia1 type isolates, indicating a relatively higher level of genetic variation within the 3' UTR than the 5'UTR.

### Phylogenetic analysis

**Figure 1:**
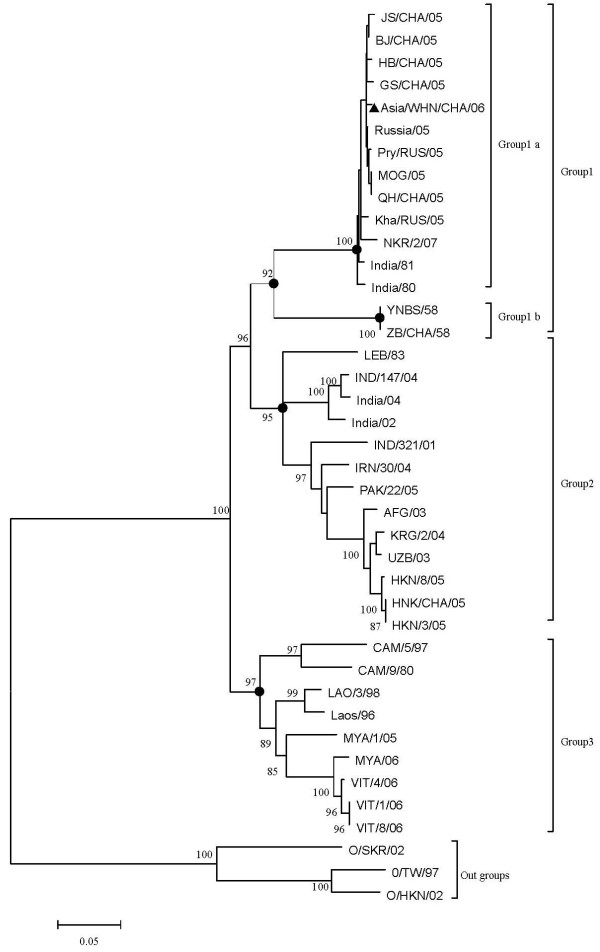
**Phylogenetic tree based on VP1 gene sequences of Asia1/WHN/CHA/06 and selected 36 reference strains**. Three sequences of VP1 gene of type O FMDV were used as outgroups.

Phylogenetic trees were constructed based on the sequence alignment of VP1 gene of 39 FMDV strains. In the phylogenetic tree, FMDV Asia1 type strains were distributed into three different groups (Figure 1). Group1 fell into two distinct groups (1a and 1b). Asia1/WHN/CHA/06 was distributed into group1a with India/80, India/81, and other 10 isolates during 2005-2007 from East Asia. Group 1b was made up of YNBS/58 and ZB/CHA/58, which were both isolated from China in 1958. Group2 contained the isolates from India, Central Asia, and Hong Kong in 1983-2005. And group3 was exclusively formed by Southeast Asia strains from 1980 to 2006.

### Recombination analysis

**Figure 2:**
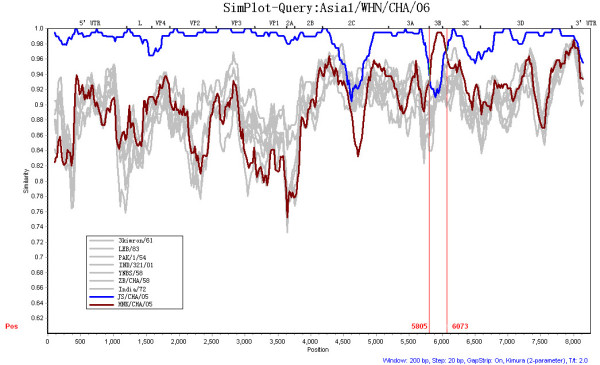
**Nucleotide similarity comparison of the complete sequences of Asia1/WHN/CHA/06 with those of representative FMDVs**. It showed a window size of 200 nt and a step size of 20 nt. The x-axis indicates nucleotide positions along the alignment and the y-axis denotes the similarity. The vertical lines show the recombination points at position 5805 and 6073.

**Figure 3:**
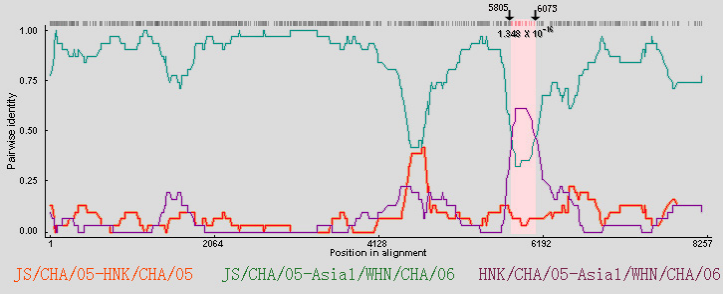
**RDP screenshots displaying the possible recombination events associated with Asia1/WHN/CHA/06**. The y-axis indicates the pairwise identity that refers to the average pairwise sequence identity within a 30nt sliding window moved one nucleotide at a time. The area outlined in pink demarcates the potential recombination regions. Crossover sites were indicated by arrows with nt positions above.

**Figure 4:**
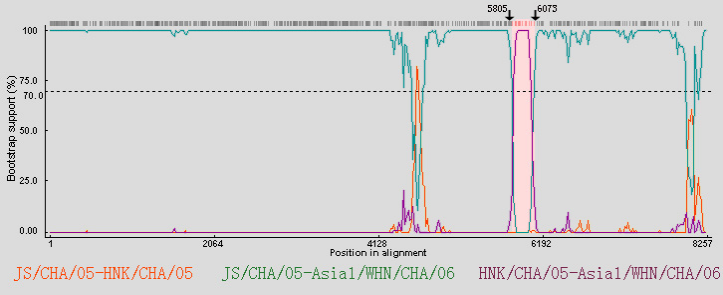
**Bootscan plots showing the likehood of recombinant sequence (Asia1/WHN/CHA/06) with JS/CHA/05 and HNK/CHA/05**. The y-axis indicates the percentage of bootstrap values that support the clustering of Asia1/WHN/CHA/06 with the parental strains. Boot strap values over 70% are considered significant. The area outlined in pink demarcates the potential recombination regions. Crossover sites were indicated by arrows with nt positions above.

In the similarity plot, Asia1/WHN/CHA/06 was shown to be closer to JS/CHA/05 than the other 8 strains. However, there were two narrow zones displaying disproportionately low levels of similarity between the two strains compared to the other regions (Figure 2). Moreover, Asia1/WHN/CHA/06 was shown to have a higher similarity to HNK/CHA/05 than JS/CHA/05 within the right narrow zone (corresponding to 3B/3C), which suggested that there was a possible recombination event in this region. And it was shown that the major parent was JS/CHA/05, and the minor parent was HNK/CHA/05. In the analysis by RDP program, the segment corresponding to 3B/3C region was defined as a crossover region (Figure 3, 4), which indicated that JS/CHA/05 and HNK/CHA/05 were possible mosaics with Asia1/WHN/CHA/06. And the estimated recombination breakpoint was at the position 5805-6073.

## 3. Disscusion

Asia1/WHN/CHA/06 was isolated from pigs in a pig farm in 2006. According to the investigation of the pig farm during the outbreak, about 20% of sows presented typical clinical symptoms of FMD, whereas none of the piglets and boars showed any symptoms and no pigs died. The FMD in the pig farm lasted for about one month. Moreover, there were not any reports about outbreak of FMD in other pig farms around the area at that time. Therefore, we speculated that Asia1/WHN/CHA/06 had low pathogenicity or infectivity to pigs, though high lethality of the strain to sulking mouth was observed in this study. But further study should be carried out to test the virulence of the strain to pigs to approve the inference.

The complete genome sequence of Asia1/WHN/CHA/06 was determined, except Poly(C) tract. Poly(C) tract of FMDV is usually ranging from 100 to 420 cytosine residues interspersed with an occasional uracil residue [19], so it is difficult to amplify the true sequence of the tract by RT-PCR from the viral RNA genome [20]. In this study, we didn't manage to get the poly(C) tract sequence of Asia1/WHN/CHA/06 either. Pseudoknots (PKs) have been found to be 3-4 tandem repeats of RNA secondary structure in FMDV genome, which probably had some role during viral replication [21]. Additionally, it is reported that partial deletions of FMDV strains in PKs' region correlated with the host range [22]. However, no deletions occurred in PKs' region of Asia1/WHN/CHA/06. In sequence of VP1 protein, the RGD motif is the molecular basis of binding of FMDV to its cellular receptor [23]. Although the RGD bordering region sequence of Asia1/WHN/CHA/06 is highly consistent with the reference strains isolated after the year of 2004, an amino acids' position at RGD +9 mutated (N→D). As several lines of evidence show that the vicinity of the RGD motif, including RGD +1 (leucine) and +4 (leucine) positions, are necessary for the FMDV RGD-mediated receptor recognition [24,25], it is unclear whether the variation at RGD +9 site influence the receptor recognition of Asia1/WHN/CHA/06. Although non-structural proteins which play important roles for viral propagation are generally more conserved than the structural proteins, 3A is one of the most variable proteins of FMDV [11]. In addition, deletions in 3A have been shown to associate with FMDV attenuation in cattle and high virulence for pigs [21]. Meanwhile, a single amino acid mutation at position 44 in the 3A protein is present in guinea pig-adapted isolates examined [26]. In our study, we found no deletion but 3 amino acid substitutions in the 3A region of Asia1/WHN/CHA/06. Considering that the isolate isolated from a pig, while other FMDV Asia1 type isolates came from cattle, it would be interesting to examine by reverse genetics if the 3 amino acid substitutions may contribute to adaptation of host range.

From a practical point of view, it is important to analyze the nucleotide sequence of the virus from new hosts for subsequent comparison with other isolates to trace the origin and route of the viral spread. To date, there are over 40 complete genome sequences for FMDV Asia1 isolated from cattle available in Genbank, but no data are available on pigs. The present study reported that the complete nucleotide sequence of Asia1/WHN/CHA/06 was isolated from pigs in southwest of China in 2006. In general, the complete genome sequence of Asia1/WHN/CHA/06 exhibited a high level of similarity with JS/CHA/05 strain, which indicated that Asia1/WHN/CHA/06 had a close genetic relationship with JS/CHA/05 strain. Phylogenetic analysis of the virus VP1 region of FMDV has been used extensively to investigate the molecular epidemiology of the disease worldwide [27]. In this study, phylogenetic tree based on VP1 region of Asia1 isolates identified the existence of three groups. Group1a mainly consisted of 11 isolates, which collected in East Asia (China, East of Russia, East of Mongolia, and North Korea) during 2005-2007. However, India/80 and India/81, which were both collected from India in 1980s, also clustered with Group1a. These data suggest that the East Asia strains, including Asia1/WHN/CHA/06, have a close genetic relationship with the two India strains emerged two decades ago.

Recombination plays an important role in FMDV evolution [28,29]. In this study, supported by Simplot and RDP program, Asia1/WHN/CHA/06 was found to be a mosaic between JS/CHA/05 and HNK/CHA/05, whose breakpoint encompassed partial 3B and 3C genes. Protein 3B, which is present in three similar but nonidentical copies (3B_1_, 3B_2 _and 3B_3_) in FMDV [30], functions in priming picornavirus RNA synthesis [31], and the 3C protease is responsible for most of the cleavages during the FMDV polyprotein processing [32]. Recombination in FMDV occurs mainly in non-structural genes [33], but the recombination over 3B/3C gene boundaries appears to be less frequent [34]. Thus, the role of the mosaicism observed in 3B/3C of FMDV should be targeted for further study. Moreover, JS/CHA/05 and HNK/CHA/05 strains were both isolated from the east of China in 2005, so there was a geographical opportunity for the recombination of the two strains. However, as Asia1/WHN/CHA/06 was isolated from west of China, it was possible that Asia1/WHN/CHA/06 was transmitted from east of China by introduction of pigs or other transmission routes.

## Conclusion

Here we reported, for the first time to our knowledge, the isolation and identification of a strain of FMDV type Asia1 from naturally infected pigs, and described the features of the genome of Asia1/WHN/CHA/06. Sequence analysis showed that it was belonged to East Asian strains, and had a close genetic relationship to JS/CHA/05. Besides, potential recombination events associated with Asia1/WHN/CHA/06 were found between JS/CHA/05 and HNK/CHA/05 strains with partial 3B and 3C fragments, which may trace the origin and evolution of Asia1/WHN/CHA/06 strain.

## Competing interests

The authors declare that they have no competing interests.

## Authors' contributions

All authors participated in the planning of the project. HNW was the leader of the project. XY and YSZ carried to the complete genome, virus isolation and identification, phylogenetic analysis and draft of the manuscript. YZ and KW participated in phylogenetic analysis and recombination analysis. TW participated in the collection of the clinical samples. All authors read and approved the final manuscript.
